# A Novel Impedimetric Microfluidic Analysis System for Transgenic Protein Cry1Ab Detection

**DOI:** 10.1038/srep43175

**Published:** 2017-03-02

**Authors:** Shunru Jin, Zunzhong Ye, Yixian Wang, Yibin Ying

**Affiliations:** 1 College of Biosystems Engineering and Food Science, Zhejiang University, Hangzhou 310058, P.R. China

## Abstract

Impedimetric analysis method is an important tool for food safety detection. In this work, a novel impedimetric microfluidic analysis system consisted of a printed gold electrode chip and a microfluidic flow cell was developed for sensitive and selective detection of transgenic protein Cry1Ab. Anti-Cry1Ab aptamer coated magnetic beads were used to recognize transgenic protein Cry1Ab and form Cry1Ab-aptamer modified magnetic beads. After separation, the obtained Cry1Ab-aptamer modified magnetic beads were dissolved in 0.01 M mannitol and followed by injection into the microfluidic flow cell for impedimetric measurement. At the frequency of 358.3 Hz, the impedance signal shows a good linearity with the concentrations of Cry1Ab protein at a range from 0 to 0.2 nM, and the detection limit is 0.015 nM. The results demonstrate that the impedimetric microfluidic analysis system provides an alternative way to enable sensitive, rapid and specific detection of transgenic protein Cry1Ab.

In recent years, exogenous *Bacillus thuringiensis* (Bt) gene is always introduced into crops by genetic modification technology to produce a serial of Bt proteins (Cry1Aa, Cry1Ab, Cry1Ac and Cry1B) to achieve the goal of insect resistance, since Bt is the most important insecticide in the field of biological control. Among the Bt proteins, Cry1Ab is one of the most common Bt proteins in genetically modified crops. However, the gradually increasing cultivation of transgenic crops has raised concerns about the ecosystem. These concerns include the harm to non-target species, geneflow, and enhancement of insect resistance. Therefore, the detection of Cry1Ab protein has become of great interest in the past decades. Several methods have been reported for the detection of Cry1Ab, such as enzyme-linked immunosorbent assay (ELISA)^1^,^2^,^3^,^4^, lateral flow immunoassay^5^, western blot method^6^, fluorescence method^7^ and surface plasmon resonance (SPR) immunosensor^8^. However, they are either labor intensive, time-consuming, require appropriate laboratory facilities and trained technicians, or suffer from low sensitivity, limited specificity and high cost. Hence, a sensitive, accurate, and rapid detection method for Cry1Ab is highly desirable.

Impedimetric biosensing method can be an ideal alternative due to its excellent advantages including high sensitivity, rapidity, ease to miniaturization, and low cost^9^,^10^,^11^, which has been used for the detection of various targets including DNA^12^,^13^, proteins^14^,^15^, pesticide^16^,^17^, heavy metal ions^18^, and bacteria^19^. In these detection methods, the bio-recognition elements, *i.e.*, DNA^20^,^21^, aptamer^22^,^23^,^24^,^25^ or antibody^26^,^27^, are immobilized on the surface of transducer to react with the targets, which produce the impedance signal^28^. Thus the related immobilization strategies play key roles, however, they suffer from some inherent drawbacks. Firstly, sensitivity is low due to the low capture efficiency of the immobilized surface for target. Secondly, reproducibility and regeneration ability are typically low. Thirdly, sequential immobilization procedures are complicated and time consuming. Therefore, the immobilization-free strategy is highly promising especially for batch and in-field applications^29^.

The application of the microfluidic technique in impedimetric biosensing is one of the important trends due to the exceptional merits in terms of sensitivity, stability, microscale bioanalysis and highthroughput^30^,^31^,^32^. Microfluidic system can improve the sensitivity of the impedance biosensor by integrating the working electrode into a microfluidic channel with a low height, by which can confine the analytes close to the electrode^33^. In addition, the microfluidic system can improve the repeatability through effectively decreasing the chances of electrode fouling, which is the major problem in microelectrode based impedance detection^34^. Finally, the microfluidic system facilitates control and manipulation of small volumes of liquid sample for impedance detection.

In this paper, we reported an aptamer based impedimetric biosensing method using the electrode immobilization-free strategy and microfluidic system for Cry1Ab protein detection. Cry1Ab protein was captured and separated by aptamer modified magnetic beads and concentrated into a desired volume with 0.01 M mannitol. The Cry1Ab-aptamer magnetic beads complexes were injected into a microfluidic flow cell with embedded printed electrode chip for impedance measurement. Compared to the previously reported methods for Cry1Ab detection, our impedimetric biosensing method exhibits higher sensitivity and shorter assay time.

## Results

### Design of the impedimetric microfluidic analysis system

**Figure 1:**
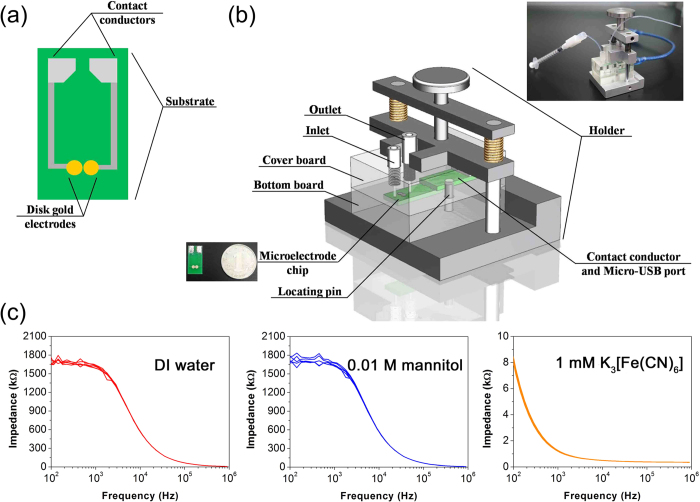
Printed electrode chip and microfluidic flow cell design. (**a**) Schematic diagram of the printed electrode chip. (**b**) Structure diagram of the microfluidic flow cell. Inset of (**b**) Photo of the impedimetric microfluidic analysis system. (**c**) The stability evaluation of the impedimetric microfluidic analysis system.Bode diagrams of impedance spectra of DI water, 0.01 M mannitol and 1 mMK_3_[Fe(CN)_6_] in the range of frequency from 100 Hz to 900 kHz.

The major problem in microelectrode based impedance detection is the signal instability due to electrode fouling or interference of the external environment. In order to solve the problem, we designed and fabricated an impedimetric microfluidic analysis system, which consists of a printed gold electrode chip and a microfluidic flow cell (Fig. 1a and b). As shown in Fig. 1a, the printed gold electrode chip consists of two disk Au electrode with diameter of 2.182 mm. Figure 1b shows the structure and photo of the impedimetric microfluidic analysis system. The impedimetric microfluidic analysis system is connected to impedance instrument using a USB data line (Inset of Fig. 1b). The microfluidic channel is above on the printed gold electrode chip with the size of 10 mm × 3.2 mm × 1 mm. The microfluidic flow cell has two polymethyl methacrylate (PMMA) components (cover board and substrate), which can be assembled and sealed with each other by the groove and plug tenon. An inlet and an outlet are punched at the two sides of the cover board. The volume of the flow channel is 32 μL, which is suitable for small amount sample detection. The stability of the impedimetric microfluidic analysis system was tested (Fig. 1c). Figure 1c shows the impedance spectra of impedimetric microfluidic analysis system in three different solvents (DI water, 0.01 M mannitol, or 1 mM K_3_[Fe(CN)_6_]) during 5 repeated cycles. The relative standard deviations (RSD) of the impedance signal of the impedimetric microfluidic analysis system at frequency of 358.3 Hz for 5 repeated cycles in DI water, 0.01 M mannitol, and 1 mM K_3_[Fe(CN)_6_] are less than 0.9%, 2.3%, and 3.2%. These results indicated that the impedimetric microfluidic analysis system has excellent stability and reproducibility.

### The affinity and specificity of Anti-Cry1Ab aptamer

**Figure 2:**
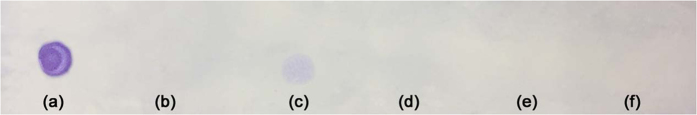
Dot blot analysis on the affinity of the aptamer with Cry1Ab. (**a**) Positive control (Avidin), (**b**) Negative control (PBS buffer), (**c**) Cry1Ab, (**d**) Cry1Aa, (**e**) Cry1Ac, (**f**) Cry1B. The concentration of Cry1Ab, Cry1Aa, Cry1Ac, and Cry1B are all 10 μM.

Dot Blot analysis was used as a conventional assay method for comparison with the biosensor method, and to observe the quality of the bio-recognition element (Anti-Cry1Ab apatmer) used in this sensor. The Dot Blot analysis procedure is shown in Supplementary Fig. S1. The target Cry1Ab and non-target Cry1Aa, Cry 1Ac and Cry1B were assayed with the Dot Blot analysis for evaluating the affinity and specificity of the Anti-Cry1Ab aptamer. As shown in Fig. 2, the aptamer only showed binding affinity to the target Cry1Ab and no binding to the non-target proteins (Cry1Aa, Cry1Ac and Cry1B), indicating good affinity and specificity of the aptamer for the target Cry1Ab.

### Fabrication of the impedimetric microfluidic analysis system for the detection of Cry1Ab

**Figure 3:**
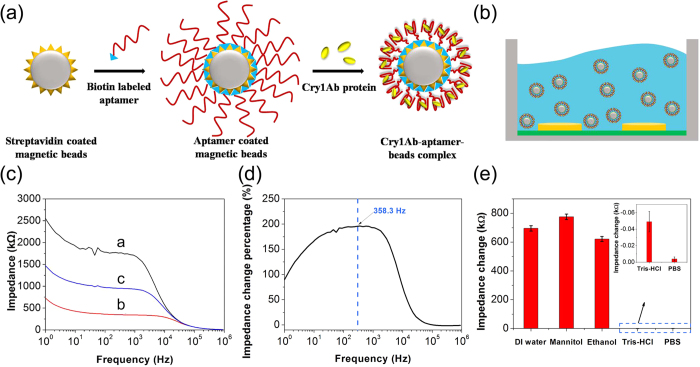
(**a**) Schematic diagram of the fabrication process of the Cry1Ab-aptamer coated magnetic bead complex. (**b**) Schematic diagram of impedance detection in the flow cell. (**c**)Bode diagram of impedance spectra for 0.01 M mannitol (curve a), aptamer modified magnetic beads suspension in 0.01 M mannitol (curve b), Cry1Ab-aptamer coated magnetic beads complexes in 0.01 M mannitol (curve c). (**d**) The difference in impedance of Cry1Ab protein binding with the concentration of 7.5 nM in the range of frequency from1 Hz to 900 kHz. (**e**) The effect on impedance detection of different solution medium including DI water, 0.01 M mannitol, 10% ethanol, binding buffer and 0.01 M pH 7.4 PBS.

The fabrication steps of Cry1Ab-aptamer coated magnetic beads complexes are shown in Fig. 3a. Biotin labeled aptamers were binding with streptavidin modified magnetic beads via the biotin–streptavidin interaction. After that step, the unbound sites were blocked with the biotins in order to avoid the nonspecific signal. Then the target Cry1Ab protein was combined with the aptamer modified magnetic beads to form the Cry1Ab-aptamer coated magnetic beads complexes, which were transferred into the 0.01 M mannitol for impedance detection (Fig. 3b). Figure 3c shows the Bode plots of impedance spectra for 0.01 M mannitol (curve a), aptamer modified magnetic beads suspension in 0.01 M mannitol (curve b), Cry1Ab-aptamer coated magnetic beads complexes in 0.01 M mannitol (curve c), respectively. Compared to the impedance value of 0.01 M mannitol, the impedance of aptamer modified magnetic beads in 0.01 M mannitol decreased significantly, because the aptamer is a single strand nucleic acid with abundant negative charges on its phosphate backbones. In our experiment, the conductivity of 0.01 M mannitol is 2–3 μS cm^−1^. When aptamer coated magnetic beads were suspended in 0.01 M mannitol with such low conductivity, they could increase the conductivity of the suspension due to their surface charges, which contribute to the decrease of the impedance value measured. The Cry1Ab-aptamer coated magnetic bead complexes showed an increase in impedance value due to the electrical nature of Cry1Ab surface^36^,^37^.

To choose the best representative frequency, we compared the impedance change between the Cry1Ab-aptamer coated magnetic beads complexes (7.5 nM Cry1Ab protein) and the aptamer modified magnetic beads (represented by (Z_sample_ − Z_control_)/Z_control_ × 100%) in the frequency range from 1 Hz to 900 kHz. Figure 3d showed that the maximum difference in the percent of impedance change was at 358.3 Hz. Thus the frequency at 358.3 Hz was chose as the testing frequency for the further experiments in this work.

In order to choose the best solution medium, we compared the impedance change between the Cry1Ab-aptamer coated magnetic beads complexes (7.5 nM Cry1Ab protein) and the aptamer modified magnetic beads (represented by Z_sample_ − Z_control_) in the different solution mediums under the optimal testing frequency (358.3 Hz). As the Fig. 3e shown, the 0.01 M mannitol got the largest impedance change signal. The Cry1Ab-aptamer modified magnetic beads complexes in binding buffer and PBS did not cause an obvious impedance change signal due to the high conductivity of binding buffer and PBS. Based on this result, we chose 0.01 M mannitol as the solution medium for the further detection experiments.

### Analytical performance of the microfluidic impedimetric analysis system

**Figure 4:**
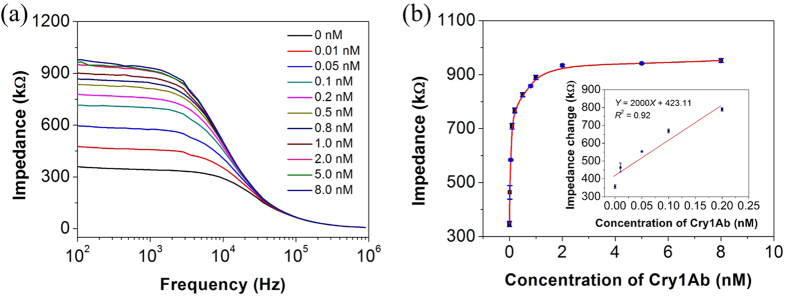
Impedimetric analysis for Cry1Ab protein. (**a**) Bode diagram of impedance spectra of the Cry1Ab-aptamer coated magnetic beads complexes in 0.01 M mannitol with the Cry1Ab concentrations of 0, 0.01, 0.05, 0.1, 0.2, 0.5, 0.8, 1, 2, 5, and 8 nM. Frequency range: 100 Hz to 900 kHz. Amplitude: 100 mV. (**b**) Calibration curve between the impedance response at the frequency of 358.3 Hz and the concentration of Cry1Ab. Inset of (**b**): The linear relationship between the impedance response at the frequency of 358.3 Hz and the concentrations of Cry1Ab.

Using the impedimetric microfluidic analysis system, bode plots for different concentrations of Cry1Ab protein (0–8 nM) were recorded. As shown in the Fig. 4a, the impedance values intensified with the increasing of Cry1Ab protein concentrations at the range from 0 to 8 nM. On the basis of the derived calibration curve (Fig. 4b), this impedimetric microfluidic analysis system shows a linear range between 0 and 0.2 nM, with a detection limit of 0.015 nM (3σ). Compared with other methods for Cry1Ab protein detection (Table 1), our analysis system shows an improved sensitivity or shorter assay time.

### The regeneration, reproducibility and specificity of the impedimetric microfluidic analysis system for Cry1Ab detection

**Figure 5:**
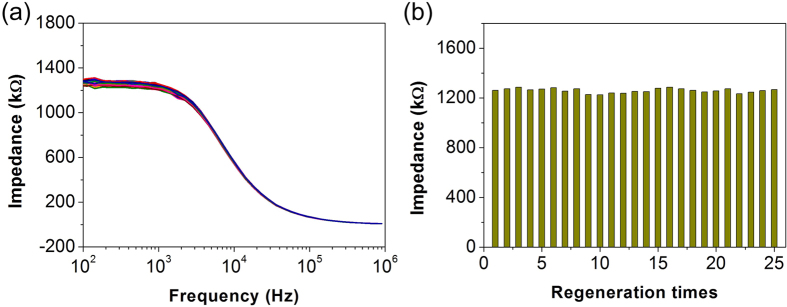
Regeneration condition of the impedimetric microfluidic analysis system. (**a**) Bode diagram of impedance spectra of the impedimetric microfluidic analysis system washed with DI water during 25 repeated times. (**b**) The impedance signal at the frequency of 358.3 Hz of the impedimetric microfluidic analysis system washed with DI water during 25 repeated times.

To investigate the regeneration condition, after the detection of Cry1Ab, the electrode was cleaned by injection of 1 mL DI water. The impedance signal of the impedimetric microfluidic analysis system during 25 repeated cycles was shown in Fig. 5a and b. The relative standard deviation (RSD) of the impedance signal at 358.3 Hz during 25 repeated times was found to be 1.38%. This result indicated that the electrode could be regenerated easily with DI water and repeatedly reused without impact on the performance of the microfluidic impedance analysis system.

Furthermore, the reproducibility of the impedimetric microfluidic analysis system for Cry1Ab (7.5 nM) detection was also checked. Ten Cry1Ab-aptamer magnetic beads complexes samples prepared under the same conditions were tested and the RSD is found to be 2.31%, indicating that impedimetric microfluidic analysis system for Cry1Ab detection is highly reproducible. And the impedance test results of 7.5 nM Cry1Ab were shown in Supplementary Fig. S2.

The non-specific transgenic proteins including Cry1Aa, Cry1Ac and Cry1B were chosen for investigating the specificity of the microfluidic impedimetric detecting method. As shown in Supplementary Fig. S3, the impedance change signal for the target protein Cry1Ab is much higher than that of non-specific proteins (Cry1Aa, Cry1Ac and Cry1B) with the same concentration, which indicated the excellent specificity of the impedimetric microfluidic analysis system for Cry1Ab detection.

### Real sample analysis

**Figure 6:**
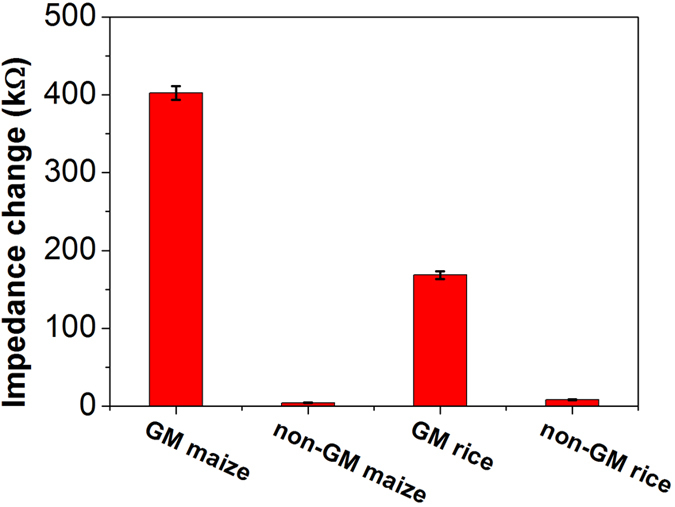
Impedance response of four crop samples at the frequency of 358.3 Hz.

The developed impedimetric microfluidic analysis system was applied to the analysis of the transgenic protein Cry1Ab in the leaves of genetically modified (GM) maize and rice. The diluted supernatant of four crop samples (GM maize, GM rice, non-GM maize, and non GM rice) was detected by this analysis system and the impedance signal at the frequency of 358.3 Hz was shown in Fig. 6. The impedance response values of GM maize and GM rice are much higher than the non-GM maize and non-GM rice, indicating that the impedimetric microfluidic analysis system can easily distinguish the GM crop and non-GM crop. Table 2 shows the concentrations of Cry1Ab in GM maize and GM rice samples measured by the impedimetric microfluidic analysis system and ELISA methods. The comparison analysis results demonstrated that the developed method had an acceptable quantitative accuracy comparing with ELISA method. All the above mentioned results indicated that the developed impedimetric microfluidic analysis system can be used for the detection of GM crop sample.

## Discussion

This research focuses on detecting the transgenic protein Cry1Ab based on microfluidic impedimetric analysis method by using aptamer modified magnetic beads as bio-recognition element. Based on this analysis method, the impedance signals at the optimal test frequency (358.3 Hz) have a good linearity with the concentrations of transgenic protein Cry1Ab at the range from 0 to 0.2 nM and an excellent detection limit of 0.015 nM. The microfluidic impedimetric analysis system with a high sensitivity effectively solved the problem of poor reproducibility and low stability in the conventional electrochemical impedimetric analysis method. Other than transgenic protein Cry1Ab, this microfluidic impedimetric analysis system also shows a great potential for the detection of other hazardous substance in food and agricultural products.

## Methods

### Reagents and materials

The biotin labeled anti-Cry1Ab protein aptamer^35^ was synthesized by Sangon Biotechnology Co., Ltd (Shanghai, China) and had the following sequence: 5′-biotin-CCGCGG-TTCCTC-GGCCCC-CTATCC-ACGCCG-AGTCCC-GAATAC-TCCCCA-GATGTA-GTAGCC-CTCAGC-ATAG-3′. The transgenic proteins (Cry1Ab, Cry1Aa, Cry1Ac, and Cry1B) were purchased form M. P. Carey CWRU (Cleveland, Ohio, USA). The avidin was purchased from Bio Basic Inc. (Markham, CA). The nitrocellulose membrane (NC membrane) with pores of 0.45 μm was purchased from Bio-Rad (Hercules, CA, USA). The streptavidin coated magnetic beads (2.8 μm in diameter) were obtained by Invitrogen (Carlsbad, CA). Non-protein blocking reagent and W-3,3,5,5-tetramethylbenzidine (TMB) chromogenic reaction kit were obtained from Sangon Biotechnology Co., Ltd (Shanghai, China). Casein and mannitol were purchased from Sigma-Aldrich (St. Louis, MO, USA). All other reagents were of analytical reagent grade. Binding solution was Tris buffer (20 mM Tris–HCl, 140 mM NaCl, 5 mM KCl, 1 mM MgCl_2_, 1 mM CaCl_2_, pH 7.4). Washing solution was PBST buffer (0.01 M PBS, 0.05% Tween 20, pH 7.4). Blocking solution was non-protein blocking reagent containing 10% casein. The biotin labeled aptamer was diluted with binding solution. Prior to use, the aptamer solution was thermally treated at 95 °C for 10 min, followed by cooling at 4 °C for 10 min. The Cry1Ab protein was dissolved in the binding solution to various concentrations. Millipore Milli-Q water (18.2 ΩM•cm) was used throughout.

### Instrument

A Solartron Analytical model 1260 Impedance-Gain-Phase Analyzer combining with a model 1287 Electrochemical Interface (Solartron Analytical, Farnborough, UK) was used for the impedance measurements. For all impedance measurements, a sine-modulated AC potential of 100 mV was applied and the impedance spectroscopy was obtained at the frequency range from 1 Hz to 900 kHz. A magnetic stand (Promega, Madison, USA) was used for all magnetic separation procedures. Injection syringes (1 mL) were obtained from Longde Medical Products Co., Ltd (Hangzhou, China).

### Printed electrode chip and microfluidic flow cell design

The microfluidic system consisted of a printed electrode chip and a microfluidic flow cell which was formed in a polymethyl methacrylate (PMMA). The printed electrode chip (Cu/Au of 35/0.15 μm) was constructed on a commercial printed circuit boards (PCB) using electrolessnickel immersion gold process (ENIG). The space of the two disk gold electrodes (2.182 mm in diameter) on the printed electrode chip was 250 μm. PMMA cover was made by patterning a computerized numerical control machine (CNC).

### Dot blot analysis

5 μL of Cry1Ab, Cry1Aa, Cry1Ac and Cry1B with same concentration of 10 μM were spotted onto the surface of the test strip (NC membrane), respectively. 5 μL of Avidin and PBS buffer were used as positive control and negative control, respectively. After the strip dried, the blocking solution was overspread onto the strip and incubated for 2 h.The strip was washed two times with washing solution and air dried. The biotin labeled aptamer (1 μM) was added to the strip with incubation for 1 h.After washing three times with washing solution, the strip was reacted with avidin-HRP (1:800 dilution) for 30 min. Excess enzyme was removed by three times washes with washing solution. Finally, the W-TMB substrate was added to the strip with incubation in the dark for 15 min at 37 °C for color development.

### Fabrication of impedimetric microfluidic analysis system for the detection of Cry1Ab protein

20 μL of streptavidin coated magnetic beads were washed using 200 μL of DI water for three times. And then biotin labeled aptamer solution (120 μL, 0.2 μM) was incubated with streptavidin coated magnetic beads for 1 h at room temperature to obtain aptamer modified magnetic beads. After washing three times with 500 μL of binding buffer, the aptamer modified magnetic beads was reacted with Cry1Ab protein (300 μL, 0–8 nM) for 1 h. After that, the Cry1Ab-aptamer-magnetic beads complex was washed for two times with 300 μL of DI water and concentrated into mannitol (200 μL, 0.01 M) for impedance testing. The Cry1Ab-aptamer-magnetic beads complex was injected into microfluidic flow cell integrated with printed electrode chip by injection syringe, and the impedance curve was collected. The impedance value at 358.3 Hz was set as the final signal.

### Determination of Cry1Ab in the fresh leaves of genetically modified (GM) maize and rice

The fresh leaves of Cry1Ab-positive genetically modified (GM) maize and rice and non-GM maize and rice (as negative control) were obtained from the Institute of Insect Sciences, Zhejiang University, China. The 0.1 g of crop leaves were cut into pieces, and then the pieces were freeze in liquid nitrogen for 2 min. After grinded into powder with TissueLyser, 500 μL of the PBS (pH7.4) was added into the sample. The obtained sample was shaken up and centrifuged (12,000 rpm, 15 min). The supernatant was diluted with binding buffer and measured using impedimetric microfluidic analysis system and Qualiplate Kit (Envirologix, Portland, Maine, USA).

## Additional Information

**How to cite this article**: Jin, S. *et al*. A Novel Impedimetric Microfluidic Analysis System for Transgenic Protein Cry1Ab Detection. *Sci. Rep.* **7**, 43175; doi: 10.1038/srep43175 (2017).

**Publisher's note:** Springer Nature remains neutral with regard to jurisdictional claims in published maps and institutional affiliations.

## Supplementary Material

Supplementary Information

## Figures and Tables

**Table 1:** Comparison study between the impedimetric microfluidic analysis system and other methods for Cry1Ab protein detection.

Method	Detection limit	Detection time	Advantage(s)	Disadvantage(s)	Ref.
ELISA	0.4 ng mL^–1^	3.5 h	High sensitivity	Time consuming, high rate of false positives	3
Fluorescence method	3 ng mL^−1^	—	Real-time, both *in vitro* and *in vivo*	High cost	7
Lateral flow immunoassay	10 pg mL^−1^	10 min	High sensitivity,	High rate of false positives	5
Surface plasmon resonance (SPR) immunosensor	4.8 ng mL^−1^	20 min	Label free, real-time, good specificity	High cost, unsuitable for fast-field analysis	8
Western blot method	2.0 ng mL^−1^	2 d	Good stability and sensitivity	Difficult to quantify, time consuming	38
Electrochemical impedance microfluidic analysis	0.015 nM (0.96 ng mL^−1^)	1 h	Label free, high sensitivity and specificity, less sample consume, good reproducibility	The detectability in real samples with the complicated matrix will be needed for further research	This work

**Table 2:** Comparsion results of the impedimetric microfluidic analysis system and the standard ELISA method for the detection of Cry1Ab protein in genetically modified (GM) maize and rice.

Samples	ELISA (nM)	This method (nM)	
GM maize	No. 1	12.00	12.60
	No. 2	10.40	11.20
	No. 3	10.80	11.40
GM rice	No. 1	3.30	3.41
	No. 2	3.25	3.35
	No. 3	2.80	3.00
