# Supplementary material for: A Novel Impedimetric Microfluidic Analysis System for Transgenic Protein Cry1Ab Detection
Source: Sci Rep. 2017 Mar 2;7:43175. doi: 10.1038/srep43175 (PMC5333080; doi:10.1038/srep43175)
Supplement: Supplementary Information [file srep43175-s1.pdf]

A Novel Impedimetric Microfluidic Analysis System for Transgenic Protein Cry1Ab Detection

Shunru Jin, Zunzhong Ye, Yixian Wang, Yibin Ying*

College of Biosystems Engineering and Food Science, Zhejiang University, Hangzhou 310058, China

**Corresponding Author. Yibin Ying, Phone& FAX: 086-571-88982885, Email: yingyb@zju.edu.cn*

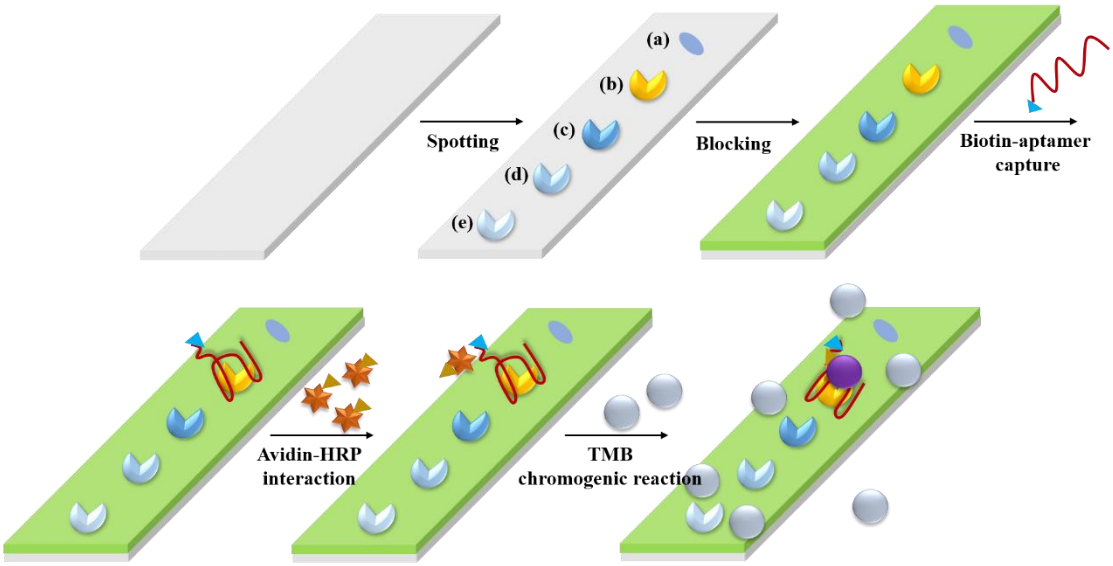

Figure S1. Schematic of the aptamer based Dot Blot analysis.

(a) (b)

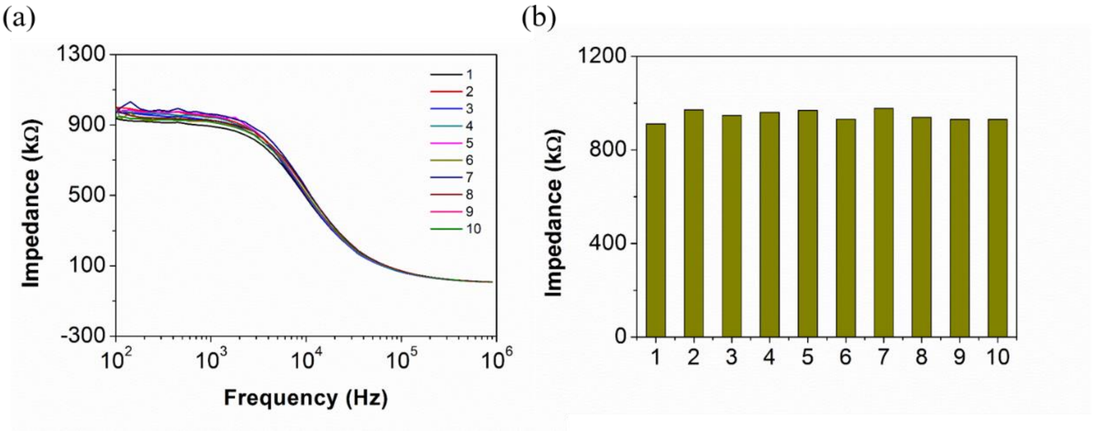

Figure S2. Reproducibility of the impedimetric microfluidic analysis system for Cry1Ab (7.5 nM) detection. (a) Bode diagram of impedance spectra of the Cry1Ab-aptamer magnetic beads complexes samples during ten repeated times. (b) The impedance signal at the frequency of 358.3 Hz of Cry1Ab-aptamer magnetic beads complexes samples during ten repeated times.

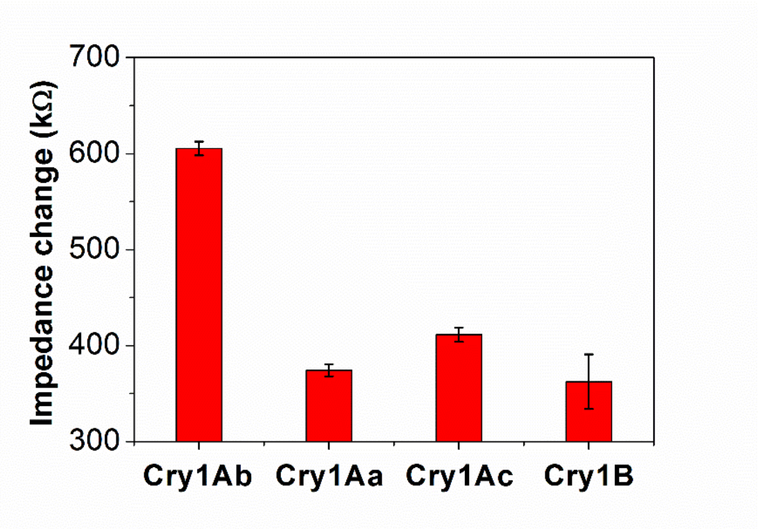

Figure S3.The specificity evaluation of this impedimetric microfluidic analysis system for Cry1Ab. The concentration of Cry1Ab, Cry1Aa, Cry1Ac, and Cry1B were all 10 nM.
